# The FORGE AHEAD clinical readiness consultation tool: a validated tool to assess clinical readiness for chronic disease care mobilization in Canada’s First Nations

**DOI:** 10.1186/s12913-017-2175-6

**Published:** 2017-03-23

**Authors:** Mariam Naqshbandi Hayward, Selam Mequanint, Jann Paquette-Warren, Ross Bailie, Alexandra Chirila, Roland Dyck, Michael Green, Anthony Hanley, Jordan Tompkins, Stewart Harris, On behalf of the FORGE AHEAD Program Team, Stewart Harris, Ed Barre, Onil Bhattacharyya, David Dannenbaum, Keith Dawson, Roland Dyck, Jo-Ann Episkenew, Michael Green, Anthony Hanley, Barry Lavallee, Ann Macaulay, Alex McComber, Monica Parry, Sonja Reichert, Jon Salsberg, Amardeep Thind, Sheldon Tobe, Ellen Toth, Audrey Walsh, Jay Wortman, Lloy Wylie, Merrick Zwarenstein, Ross Bailie, Kayla Collins, Claire de Oliveira, Michael Hindmarsh, Valeria Rac, Linda Stanley, Joanne Lewis, Marlene Nosé, Brigitte Parent, Stephen Sundquist, Lillian Houle, Amber Houle, Dawn Montour-Lazare, Joelle Emond, Jessica Jacobs, Randy Littlechild, Bonny Graham, Tina Littlechild, Devon Guy, Chalsea Onespot, Ivan Kimble McComb, Emilie Dufour, Verna Jolly, Charlene Diamond, Jennifer Jones, Danna Hadden, April DeYaeger, Theresa O’Keefe, Ada Roberts, Maggie Organ, Shelley Kirkness, Marie Jebb, Carla Constant, Allen Deleary, Rennie Nawash, Lori Sinclair, Heather McDonald, Bonnie Nickel, Mariam Naqshbandi Hayward, Selam Mequanint, Jann Paquette-Warren, Jordan Tompkins, Susan Webster-Bogaert, Braden Te Hiwi, Harsh Zaran, Jim Esler, Meghan Fournie, Marie Tyler, Jackie McLellan, Marnie Orcutt

**Affiliations:** 1 Centre for Studies in Family Medicine, Western Centre for Public Health and Family Medicine, Department of Family Medicine, Schulich School of Medicine and Dentistry, Western University, 1151 Richmond Street, London, Ontario N6A 3K7 Canada; 2 Menzies School of Health Research, Charles Darwin University, Darwin, Northern Territory Australia; 3 Canadian Center for Health and Safety in Agriculture, Department of Medicine, College of Medicine, University of Saskatchewan, Saskatoon, Saskatchewan Canada; 4 Departments of Family Medicine and Public Health Sciences, Queen’s University, Kingston, Ontario Canada; 5 Departments of Nutritional Sciences and Medicine and the Dalla Lana School of Public Health, University of Toronto, Toronto, Ontario Canada

**Keywords:** Readiness, Diabetes mellitus, Quality improvement, Chronic disease, Indigenous, First Nations

## Abstract

**Background:**

Given the astounding rates of diabetes and related complications, and the barriers to providing care present in Indigenous communities in Canada, intervention strategies that take into account contextual factors such as readiness to mobilize are needed to maximize improvements and increase the likelihood of success and sustainment. As part of the national FORGE AHEAD Program, we sought to develop, test and validate a clinical readiness consultation tool aimed at assessing the readiness of clinical teams working on-reserve in First Nations communities to participate in quality improvement (QI) to enhance diabetes care in Canada.

**Methods:**

A literature review was conducted to identify existing readiness tools. The ABCD – SAT was adapted using a consensus approach that emphasized a community-based participatory approach and prioritized the knowledge and wisdom held by community members. The tool was piloted with a group of 16 people from 7 provinces and 11 partnering communities to assess language use, clarity, relevance, format, and ease of completion using examples. Internal reliability analysis and convergence validity were conducted with data from 53 clinical team members from 11 First Nations communities (3–5 per community) who have participated in the FORGE AHEAD program.

**Results:**

The 27-page Clinical Readiness Consultation Tool (CRCT) consists of five main components, 21 sub-components, and 74 items that are aligned with the Expanded Chronic Care Model. Five-point Likert scale feedback from the pilot ranged from 3.25 to 4.5. Length of the tool was reported as a drawback but respondents noted that all the items were needed to provide a comprehensive picture of the healthcare system. Results for internal consistency showed that all sub-components except for two were within acceptable ranges (0.77–0.93). The Team Structure and Function sub-component scale had a moderately significant positive correlation with the validated Team Climate Inventory, *r* = 0.45, *p* < 0.05.

**Conclusions:**

The testing and validation of the FORGE AHEAD CRCT demonstrated that the tool is acceptable, valid and reliable. The CRCT has been successfully used to support the implementation of the FORGE AHEAD Program and the health services changes that partnering First Nations communities have designed and undertaken to improve diabetes care.

**Trial registration number:**

Current ClinicalTrial.gov protocol ID NCT02234973. Date of Registration: July 30, 2014

**Electronic supplementary material:**

The online version of this article (doi:10.1186/s12913-017-2175-6) contains supplementary material, which is available to authorized users.

## Background

The burden of diabetes on both families and the healthcare system in Canada is growing at an astonishing pace with prevalence rates that have more than doubled in the last decade [1]. In 2010, 7.6% of the total population had diabetes [2]. More alarming are the widening health disparities for Indigenous peoples in Canada [3]. With rising diabetes incidence and prevalence rates [1], higher rates of gestational diabetes [4], a younger age of diabetes diagnosis [5], increasing rates of type 2 diabetes in children and adolescents [6], and higher rates of diabetes complications and comorbidities [7], Indigenous population-specific interventions for type 2 diabetes have become vital. Such health disparities between Indigenous and non-Indigenous peoples have been observed in many countries around the world, primarily in relation to chronic diseases [8, 9]. Consequently, more effective delivery of healthcare to Indigenous peoples has become an urgent priority.

It has been suggested that barriers to optimal diabetes care are different and sometimes more pronounced in Indigenous communities in Canada than those experienced in non-Indigenous communities due to geographic isolation, cultural differences and the disjointed healthcare provided by a combination of federal government, provinces and territories [10, 11]. Given the barriers present in Indigenous communities such as availability and accessibility of primary healthcare services and professionals, appropriate and effective intervention strategies that take into account contextual factors in their planning and evaluation are needed to maximize improvements and increase the likelihood of success and sustainment [12, 13]. Readiness is a measure of the recognition, preparation, and/or action taken when facing an area that requires change or improvement [14] and has been identified as a critical contextual factor to incorporate into public health interventions in Indigenous communities [12, 15].

When evaluating readiness relative to the healthcare setting in Indigenous communities in Canada, a valuable assessment is possible when all individuals involved in the prevention and treatment process participate (patients, the community, healthcare providers, etc.). The results of these evaluations can be used to guide care and maximize health improvements [16]. Holt et al [17] identified psychological and structural factors as key elements of health teams’ readiness. Psychological factors are the beliefs an individual holds regarding change, recognition of the problem requiring change as a priority, and agreement with the change plans. Structural factors are the circumstances under which the change is being implemented such as an organization’s capabilities and resources [17]. In an extensive review of 106 peer-reviewed articles on organizational readiness for change, Weiner et al [18] concluded that the content of a comprehensive organizational readiness tool should include a psychological and structural approach at an individual and organizational level that respects both macro-level structural factors and micro-level factors (e.g., individual differences). Recently, Attieh et al’s [19] review of organizational readiness for knowledge translation in chronic care identified five core concepts, namely: organizational dynamics, change process, innovation readiness, institutional readiness and personal readiness.

Determination of health teams’ degree of readiness is advantageous with regards to maximizing the success of interventions through the identification of available resources and the present knowledge of a health issue [20]. A low readiness score, for example, may indicate that healthcare staff face substantial challenges due to inadequate support from local staff, leadership or resources when implementing an intervention or program. In such an instance, an intervention should aim to address some of these challenges in order to increase the likelihood of success. By contrast, local staff can mount more complex interventions when a high degree of readiness is accessed owing to higher levels of leadership, resources and local knowledge or expertise.

The *TransFORmation of IndiGEnous PrimAry HEAlthcare Delivery (FORGE AHEAD): Community-driven Innovations and Strategic Scale-up Toolkits* is a 5-year national research program that partners with Indigenous communities in Canada to improve chronic disease care and access to available resources by developing and evaluating community-driven, culturally-relevant primary healthcare models through a quality improvement (QI) process [21]. The program uses a participatory research approach that simultaneously ensures culturally appropriate implementation and integrates knowledge translation by involving relevant stakeholders throughout the entire program. Two QI teams, a community and a clinical team, mobilized in each partnering community are provided training and support to implement various QI initiatives. Recognizing the importance of matching QI to a clinical teams’ readiness level as essential for success and sustainment [17, 22]; and given the national scope of the FORGE AHEAD Program, a cost-effective and flexible clinical readiness consultation tool capable of identifying key factors that influence the adoption of initiatives to address chronic disease care in the unique communities in Canada was sought. We report here the development, testing and validation of the FORGE AHEAD CRCT.

## Methods

### Aim, design and setting

The objective was to develop a validated Clinical Readiness Consultation Tool (CRCT) to assess the readiness of clinical teams working on-reserve in Indigenous communities before they engage in QI efforts to enhance diabetes care in Canada. A consensus approach [23] within the community-based participatory research framework was utilized to develop and test the CRCT. Community-based participatory research emphasizes the recognition of a community as an equal partner and stakeholder that collaborates in all phases of the research [24]. Actions and decisions are made in a co-learning and knowledge sharing domain that facilitates research built on community strengths and adapts to community priorities to maximize both researcher and community benefits [24, 25]. The emergence of a co-operative model for health research and practice based on consensus with community partners is not new. The idea of working *with* communities rather than *on* communities is consistently recognized as a critical factor in research with Indigenous partners [23, 24, 26, 27]. The consensus approach based on the community-based participatory research framework differs from traditional approaches such as the Delphi process where emphasis is placed on expert knowledge and advanced detailed planning. Such traditional approaches have been documented as inappropriate when conducting health research with Indigenous community partners [24].

FORGE AHEAD partners with 11 First Nations communities across 6 provinces (BC, AB, MB, ON, QC, NL) and three isolation levels (isolated, non-isolated, and remote-isolated/semi-isolated).

### Development of the Clinical Readiness Consultation Tool (CRCT)

A literature review was conducted in March 2014 using PubMed to identify existing clinical readiness tools published prior to January 2014. Key search terms included “Indigenous” or “First Nations” or “Aboriginal AND “healthcare” or “primary healthcare” or “chronic disease” AND “clinical readiness”, or “clinical change readiness” or “organizational readiness” or “organizational change” or “change management”. With no tools found in the Canadian Indigenous domain, the search was expanded internationally, included all populations, and examined grey literature for key content. A total of 13 PubMed articles and four grey literature articles/sources were found. Inclusion criteria included use in Indigenous populations, a health domain tool, and restricted to clinicians (versus patients).

Members of the Working Group which consisted of experts in the fields of readiness, Indigenous research, survey development and chronic disease care/epidemiology assessed the literature to identify the most appropriate tools for use within FORGE AHEAD. From the review of literature, the Chronic Care Model [28], Expanded Chronic Care Model [29], Assessment of Chronic Illness Care (ACIC) tool [30] and the Audit and Best Practice for Chronic Disease – Systems Assessment Tool (ABCD-SAT) [31, 32] were identified as key tools that examined organizations for strengths and areas to target for improvements or degree of readiness. The Chronic Care Model and the more health-promotion focused Expanded Chronic Care Model are both conceptual frameworks that guide implementation of chronic care, but cannot be used as practical tools to assess healthcare team readiness to address chronic disease change [33]. The ACIC and the ABCD-SAT, adapted from the ACIC to fit the local Australian setting, were both based on the concepts of the Chronic Care Model and emphasize system-level factors in the health setting for chronic disease change. Compared with the original ACIC scale, the adapted ABCD-SAT included three additional items (i.e., cultural competence, pathology management, and pharmacy management) [32]. The ABCD-SAT has been used extensively in Indigenous communities and health services in Australia, was designed to understand the state of development of health centre systems and inform decisions regarding priority areas for system improvement [32]. The ABCD-SAT is administered to a group of healthcare providers (managers, nurses, physicians) by external researchers or an external facilitator who guides the group through the components in the tool. The facilitator has been recognized as an integral part of using the ABCD-SAT with clinics who have demonstrated a level of engagement, interest and commitment to quality improvement.

The aforementioned existing tools (Chronic Care Model, Expanded Chronic Care Model, ACIC, and ABCD-SAT) were considered for the FORGE AHEAD program; however the following drawbacks were identified: i) reliance on costly resources external to the community for tool implementation and/or interpretation, ii) not tailored to the unique Canadian setting and First Nations primary healthcare setting, iii) not focused on type 2 diabetes, iv) lack of a qualitative component where Indigenous team members’ can add information regarding their insights and perceptions regarding diabetes care, and, v) no built-in mechanism to integrate individual perceptions to be used to support clinical readiness at a small group level. Therefore, the FORGE AHEAD Program aimed to develop and test a CRCT relevant to diabetes care in the Canadian Indigenous context that could be applied by primarily using existing internal community resources without the need for external interviewers and transcription. The ABCD-SAT (Version 2) was selected by the Working Group as the basis for conceptualizing and developing the CRCT. The overall goal of the Working Group was to develop a comprehensive, user-friendly, understandable, and stand-alone questionnaire that could be administered and used by community-based facilitators (rather than external interviewers) in First Nations communities within the FORGE AHEAD Program to assess readiness to adopt chronic disease interventions.

Adapting the ABCD-SAT into the CRCT involved a thorough review by Working Group members over a series of six rounds from January to July 2014. Each of the components and sub-components as well as the individual items that encompass the sub-components of the ABCD-SAT were reviewed for relevance to the context of Canadian First Nations primary healthcare delivery systems, redundancies, cultural relevance, and language. One item was added on cultural competence and two questions about registries were removed. Type 2 diabetes specific examples were inserted to enhance clarity in most items. All items were revised to reflect the Canadian First Nations context and the language was adjusted for improved clarity and ease of understanding.

### Pilot testing

In June 2014, 15 people from 7 provinces comprised of 3 co-investigators and 12 Indigenous and non-Indigenous community members from 11 partnering communities received the pilot CRCT. This group was selected given their prior involvement in the planning and development of the FORGE AHEAD Research Program proposal. Community members included family physicians, nurses, health managers and administrators, health promoters, and adult educators. The draft CRCT was distributed electronically along with a feedback form (see Additional file 1) that was designed to gather information on a five-point Likert scale on how respondents perceived the following: (1) appropriate language use for First Nations health clinics in Canada, (2) clarity of questions, (3) relevance of questions to health clinics in First Nations communities, (4) appropriate format for the tool, and, (5) helpfulness of examples provided for each question to complete the clinical assessment tool. A qualitative (open-ended questions) section asked respondents for suggestions, comments and specific recommendations. Respondents were asked to complete the questionnaire and insert comments in the questionnaire, as well as fill in the feedback form. To summarize the data, scores were tallied for each of the five scored domains and short responses and suggestions for improvements were summarized. All feedback received was incorporated into the revisions. The CRCT was deemed acceptable in each domain if a score of 3 or higher was received and if qualitative feedback did not raise concerns.

### Statistical validation of the tool

To evaluate the technical quality of the CRCT, internal reliability analysis and convergence validity were conducted. Individuals involved in patient care from each of the FORGE AHEAD partnering communities were asked to complete the CRCT as part of the program intervention. A total of 53 clinical team members from 11 First Nations communities (3-5 FORGE AHEAD program participants per community) completed the CRCT at three time points: pre, during, and post program implementation. For this study, only pre-program implementation data was used to avoid a learning effect bias.

The internal reliability assessment was based on the correlations between the individual items that make up the sub-components, relative to the variances [34]. The internal consistency of each sub-component was calculated using Cronbach’s alpha (*α*) which measures whether a group of items is related to a single construct or sub-component [35]. The higher the average correlation among the items, the higher the *α* but an *α* greater than 0.9 may also be indicative of a redundant item. An *α* greater than or equal to 0.7 is considered satisfactory (34). Sub-components with a single item were excluded from the internal consistency analysis. Overall *α* for the total scale (CRCT) was not calculated because each sub-component is intended to measure a distinct factor. Missing data was handled through pairwise deletion (available-case analysis) of cases. Descriptive statistics were generated to evaluate the score distribution and proportion of missing data per item. Items with high levels of missing data (>10%) were identified and considered for further analysis and interpretation.

As part of FORGE AHEAD, participants also completed the Team Climate Inventory (TCI) to examine team functioning of clinical teams working on-reserve. The TCI (19 items divided into four subscales with either a 5 or 7 point-Likert scale) has been validated [36] and used in a variety of contexts [37]. The CRCT includes a sub-component that measures Team Structure and Function (TSF) with five 12 point-Likert scale items. Convergence validity analysis was carried out assessing the correlation between the scores of the TCI and TSF to evaluate the degree to which these tests assess the same construct [38]. Data was used in this analysis only when both tools were completed by the same individual during the same time period (pre-program implementation). The total score for each individual scale was calculated for each participant who completed both questionnaires. Pearson correlation, appropriate for interval scale data [39] was used to assess convergence validity. Statistical Analysis Software (SAS) version 9.2 was used to conduct the validation analysis.

## Results

### FORGE AHEAD Clinical Readiness Consultation Tool (CRCT)

The 27-page CRCT (see Additional file 2) has 4 main sections: 1) 1-page introduction describing the background, confidentiality, benefits, risks, reimbursement, consent, and contact information; 2) 1-page brief instruction (estimated time to complete, brief description of rating scales and how to submit the completed questionnaire); 3) general information (brief 8-item demographic profile); and 4) 5 main components and sub-components of healthcare systems important in chronic disease care (aligned with Expanded Chronic Care Model). The questionnaire is designed to be anonymous and provides a section to insert participant numbers.

A total of 74 items comprise the CRCT. Data on the general demographic characteristics of respondents were collected using the first 8 items. The rest of the items are categorized into 5 major components and a total of 21 sub-components (Table 1). The 5 components include Delivery System Design, Information Systems and Decision Support, Self-management Support, Linkages with Community Resources and Other Health Services, and Local Health Center Organizational Influence and Integration. Each sub-component consists of between 1 and 6 items and often includes an example. Items were rated on a four category 12-point Likert scale defined as ‘limited or no support’ (score 0–2), ‘basic support’ (score 3–5), ‘good support’ (score 6–8), and ‘fully developed support’ (score 9–11). For each category, brief descriptions guide participants to consider the score that best represents their clinical context. A justification box was provided below each item/rating scale for participants to explain their rating of provided written comments. Responses by each clinical team participant are calculated and summarized in an aggregated group report.

**Table 1:** Clinical Readiness Consultation Tool (CRCT) structure

Clinical Readiness Consultations: Components and Sub-components	# Items
Component 1: Delivery System Design (28 items)	
1.1 Team Structure and Function	5
1.2 Clinical Leadership	3
1.3 Appointments and Scheduling	3
1.4 Care Planning	2
1.5 Systematic Approach to Follow-up	4
1.6 Continuity of Care	2
1.7 Patient Access	2
1.8 Cultural Competence/Knowledge	4
1.9 Physical Infrastructure	3
Component 2: Information Systems and Decision Support (6 items)	
2.1 Maintenance and Use of an Electronic or Paper Diabetes Registry	2
2.2 Evidence-based Guidelines for Diabetes	3
2.3 Specialist and Generalist Collaboration	1
Component 3: Self-management Support (7 items)	
3.1 Self-Management Support, Assessment and Documentation	3
3.2 Self-management Education, Behavioral Risk Reduction and Peer Support	3
Component 4: Linkages with Community Resources and Other Health Services (15 items)	
4.1 Communication and Cooperation of the Health Center and Other Community-based Organizations and Programs	6
4.2 Linking Health Center Patients to Community Resources	3
4.3 Community Outreach	3
4.4 Regional Health Planning and Development of Health Resources	3
Component 5: Local Health Center Organizational Influence and Integration (10 items)	
5.1 Organizational Commitment	5
5.2 Quality Improvement Strategies	4
5.3 Integration of Health System Components to Achieve High Quality Care for Patients with Diabetes	1

### Pilot testing results

A total of 8 respondents from 4 provinces (2 Co-Investigators, 6 community representatives from 6 First Nations communities) completed the feedback form for an overall review and response rate of 53.3%. The CRCT took an average of 118 min to complete and ranged from 53 to 240 min. Based on the five-point Likert scale feedback form, average respondent scores were: appropriate language use for First Nations health clinics in Canada (3.75); questions clearly written (3.25); questions are relevant to health clinics in First Nations communities (4); format of the tool is appropriate (4.5); and, helpfulness of examples to complete the clinical readiness assessment (4). The 12-point Likert scale of the CRCT was maintained based on the qualitative feedback. Analysis of the written answers to open-ended questions revealed that although the questionnaire was long, respondents found that all the questions were important and necessary to obtain a comprehensive understanding of the current services available in communities. Some respondents noted that the language was geared for higher education/reading and comprehension skills. Revisions to the tool were made following the pilot to improve clarity to address this concern.

### Statistical validation of the tool

#### Participant demographics

Of the 53 people who completed the CRCT, 87% were female and 62% were Indigenous (see Table 2 for more participant demographics).

**Table 2:** Demographic characteristics of participants

Variable	Characteristics	N (%)
Gender	Female	46(87%)
Indigenous	Yes	33(62%)
Role	Family Physician	7(13%)
	Nurse Practitioners	4(8%)
	Registered Nurse	16(30%)
	Registered Dietician	4(8%)
	Health Director/Program Managers	4(8%)
	Other (Clinical Assistances, Health Care Aid, Case Manager etc.)	18(34%)

#### Missing data

There was a high completion rate for most of the items with only 8 items from three components left unanswered by more than 10% of the participants (see Table 3). Given the relatively high number of items in the CRCT, this represented a small number of items with missing data. Table 3 further shows that the majority of participants scored items of the CRCT between 3 and 7 on the interquartile range scale, indicating that the scores are not clustered together toward the low or high end of the scale.

**Table 3:** Descriptive statistics by CRCT item

Sub-components	Items	N	Miss N (%)	Median (IQR)^a^	Min N^b^ (%)	Max N^c^ (%)
Team structure and function	TeamFunction_1_1A	52	1(1.9)	8(3.5)	1(1.9)	5(9.6)
	TeamFunction_1_1B	53	0(0.0)	5(6)	2(3.8)	2(3.8)
	TeamFunction_1_1C	51	2(3.8)	6(6)	2(3.9)	2(3.9)
	TeamFunction_1_1D	51	2(3.8)	5(5)	1(2.0)	2(3.9)
	TeamFunction_1_1E	52	1(1.9)	7.5(4)	1(1.9)	4(7.7)
Clinical leadership	Leadership_1_2A	53	0(0.0)	8(5)	1(1.9)	5(9.4)
	Leadership_1_2B	50	3(5.7)	8(4)	1(2.0)	4(8.0)
	Leadership_1_2C	50	3(5.7)	7(4)	3(6.0)	3(6.0)
Appointments and scheduling	Appointments_1_3A	52	1(1.9)	7(5)	1(1.9)	12(23.1)
	Appointments_1_3B	52	1(1.9)	8(3)	1(1.9)	7(13.5)
	Appointments_1_3C	51	2(3.8)	8(5)	2(3.9)	11(21.6)
Care planning	CarePlanning_1_4A	51	2(3.8)	6(5)	3(5.9)	1(2.0)
	CarePlanning_1_4B	51	2(3.8)	6(5)	2(3.9)	4(7.8)
Systematic approach to follow-up	Followup_1_5A	53	0(0.0)	6(4)	2(3.8)	5(9.4)
	Followup_1_5B	51	2(3.8)	9(5)	2(3.9)	9(17.7)
	Followup_1_5C	52	1(1.9)	8.5(4)	1(1.9)	11(21.2)
	Followup_1_5D	52	1(1.9)	5(8)	13(25)	4(7.7)
Continuity of care	Continuity_1_6A	52	1(1.9)	6(4.5)	5(9.6)	5(9.6)
	Continuity_1_6B	51	2(3.8)	7(5)	1(2.0)	3(5.9)
Patient access	Access_1_7A	51	2(3.8)	8.5(4)	1(2.0)	9(17.3)
	Access_1_7B	49	4(7.5)	9(5)	2(4.1)	13(26.5)
Cultural competence/Knowledge	CulturalCompetence_1_8A	51	2(3.8)	8(5)	2(3.9)	6(11.8)
	CulturalCompetence_1_8B	46	7(13.2)	9(3)	3(6.5)	14(30.4)
	CulturalCompetence_1_8C	50	3(5.7)	6(5)	3(6.0)	7(14.0)
	CulturalCompetence_1_8D	51	2(3.8)	9(5)	1(2.0)	13(25.5)
Physical infrastructure	Infrastructure_1_9A	53	0(0.0)	8(5)	1(1.9)	11(20.8)
	Infrastructure_1_9B	51	2(3.8)	10(3)	1(2.0)	21(41.2)
	Infrastructure_1_9C	52	1(1.9)	8(5.5)	1(2.0)	13(25.0)
Diabetes registry	Registry_2_1A	48	5(9.4)	6(6)	5(10.4)	7(14.6)
	Registry_2_1B	48	5(9.4)	5(6)	8(16.7)	2(10.4)
Diabetes practice guidelines	EBG_2_2A	51	2(3.8)	9(4)	2(3.9)	6(11.8)
	EBG_2_2B	51	2(3.8)	9(3)	3(5.9)	8(15.7)
	EBG_2_2C	49	4(7.5)	8(5)	3(6.1)	6(12.2)
Specialist and generalist	Collaboration_2_3A	51	2(3.8)	8(6)	3(5.9)	5(9.8)
Self-management support	SM_3_1A	52	1(1.9)	8(3)	1(2.0)	3(5.8)
	SM_3_1B	52	1(1.9)	6(4.5)	5(9.6)	2(3.9)
	SM_3_1C	52	1(1.9)	7(5)	2(3.9)	6(11.5)
Self-management education	SM_3_2A	49	4(7.5)	8(4)	2(4.1)	6(12.2)
	SM_3_2B	49	4(7.5)	5(6)	3(6.1)	5(10.2)
	SM_3_2C	52	1(1.9)	6(4)	4(7.7)	4(7.7)
	SM_3_2D	53	0(0.0)	8(3)	2(3.8)	5(9.4)
Communication and cooperation	Communication_4_1A	48	5(9.4)	6.5(5)	2(4.2)	2(4.2)
	Communication_4_1B	49	4(7.5)	7(5)	1(2.0)	4(8.2)
	Communication_4_1C	49	4(7.5)	5(6)	3(6.1)	8(16.3)
	Communication_4_1D	51	2(3.8)	5(7)	6(11.8)	2(3.9)
	Communication_4_1E	48	5(9.4)	8(4)	3(6.3)	2(4.2)
	Communication_4_1F	49	4(7.5)	6(5)	1(2.0)	7(14.3)
Linking patients to community resources	LinkResources_4_2A	50	3(5.7)	7.5(4)	2(4.0)	2(4.0)
	LinkResources_4_2B	47	6(11.3)	5(7)	5(10.7)	1(2.1)
	LinkResources_4_2C	48	5(9.4)	5(6)	6(12.5)	1(2.1)
Community outreach	Outreach_4_3A	51	2(3.8)	9(4)	1(2.0)	10(19.6)
	Outreach_4_3B	47	6(11.3)	7(4)	2(4.3)	3(6.4)
	Outreach_4_3C	50	3(5.7)	7.5(4)	1(2.0)	3(6.0)
Planning and development	Planning_4_4A	42	11(20.8)	5(6)	5(11.9)	5(11.9)
	Planning_4_4B	49	4(7.5)	5(6)	5(10.2)	1(2.0)
	Planning_4_4C	43	10(18.9)	5(6)	6(14.0)	4(9.3)
Organizational commitment	OrgCommitment_5_1A	48	5(9.4)	8(4)	1(2.1)	3(6.3)
	OrgCommitment_5_1B	43	10(18.9)	7(4)	3(7.0)	5(11.6)
	OrgCommitment_5_1C	50	3(5.7)	6.5(4)	1(2.0)	5(10.0)
	OrgCommitment_5_1D	52	1(1.9)	8(4)	1(1.9)	12(23.1)
	OrgCommitment_5_1E	50	3(5.7)	8(5)	1(2.0)	8(16.0)
Quality improvement strategies	QI_5_2A	49	4(7.5)	9(3)	1(2.0)	11(22.5)
	QI_5_2B	44	9(17.0)	6.5(4)	4(9.1)	6(13.6)
	QI_5_2C	43	10(18.9)	6(6)	2(4.7)	5(11.6)
	QI_5_2D	50	3(5.7)	6(6)	6(12.0)	4(8.0)
Integration of health system	Integration_5_3A	52	1(1.9)	6(4.5)	2(3.9)	4(7.7)

^a^IQR: Interquartile range calculated by subtracting the first quartile from the quartile of the data

^b^Min N (%) represents the number (percentage) of respondents ranked an item the minimum (0) in the CRCT scale

^c^Max N represents the number (percentage) of respondents ranked an item the maximum (12) in the CRCT scale

#### Internal consistency

Two sub-components, namely, Specialist and Generalist Collaboration, and Integration of Health System, were excluded from the internal consistency analysis due to an insufficient number of items (single item) to calculate sub-component *α*. Results show that all sub-components except for two were within acceptable ranges (0.77–0.93) (Table 4). Two sub-components (Appointment and Scheduling, and Patient Access) had low (<0.7) internal consistency while seven sub-components had *α* of above 0.9.

**Table 4:** Internal consistency of 21 sub-scales of the CRCT analysed per subcomponent

Subcomponent	Items	Correlation with Total	Alpha(*α*) if deleted	*α*
Team structure and function	TeamFunction_1_1A	0.77	0.91	0.92
	TeamFunction_1_1B	0.85	0.89	
	TeamFunction_1_1C	0.90	0.88	
	TeamFunction_1_1D	0.74	0.91	
	TeamFunction_1_1E	0.73	0.92	
Clinical leadership	Leadership_1_2A	0.82	0.82	0.91
	Leadership_1_2B	0.82	0.82	
	Leadership_1_2C	0.83	0.83	
Appointments and scheduling	Appointments_1_3A	0.53	0.49	0.66
	Appointments_1_3B	0.52	0.52	
	Appointments_1_3C	0.39	0.69	
Care planning	CarePlanning_1_4A	0.80	-	0.89
	CarePlanning_1_4B	0.80	-	
Systematic approach to follow-up	Followup_1_5A	0.65	0.68	0.77
	Followup_1_5B	0.61	0.69	
	Followup_1_5C	0.63	0.68	
	Followup_1_5D	0.45	0.80	
Continuity of care	Continuity_1_6A	0.79	-	0.88
	Continuity_1_6B	0.79	-	
Patient access	Access_1_7A	0.47		0.63
	Access_1_7B	0.47		
Cultural competence/Knowledge	CulturalCompetence_1_8A	0.75	0.82	0.87
	CulturalCompetence_1_8B	0.75	0.82	
	CulturalCompetence_1_8C	0.76	0.82	
	CulturalCompetence_1_8D	0.64	0.86	
Physical infrastructure	Infrastructure_1_9A	0.59	0.64	0.74
	Infrastructure_1_9B	0.50	0.73	
	Infrastructure_1_9C	0.64	0.58	
Diabetes registry	Registry_2_1A	0.84	-	0.91
	Registry_2_1B	0.84	-	
Diabetes practice guidelines	EBG_2_2A	0.83	0.72	0.85
	EBG_2_2B	0.80	0.74	
	EBG_2_2C	0.62	0.95	
Self-management support	SM_3_1A	0.67	0.91	0.88
	SM_3_1B	0.83	0.78	
	SM_3_1C	0.83	0.77	
Self-management education	SM_3_2A	0.77	0.88	0.90
	SM_3_2B	0.81	0.87	
	SM_3_2C	0.76	0.88	
	SM_3_2D	0.81	0.87	
Communication and cooperation	Communication_4_1A	0.84	0.88	0.91
	Communication_4_1B	0.73	0.89	
	Communication_4_1C	0.83	0.88	
	Communication_4_1D	0.66	0.91	
	Communication_4_1E	0.83	0.88	
	Communication_4_1F	0.61	0.91	
Linking patients to community resources	LinkResources_4_2A	0.79	0.96	0.93
	LinkResources_4_2B	0.93	0.83	
	LinkResources_4_2C	0.89	0.87	
Community outreach	Outreach_4_3A	0.81	0.90	0.92
	Outreach_4_3B	0.82	0.88	
	Outreach_4_3C	0.86	0.85	
Planning and development	Planning_4_4A	0.78	0.74	0.85
	Planning_4_4B	0.69	0.83	
	Planning_4_4C	0.71	0.81	
Organizational commitment	OrgCommitment_5_1A	0.83	0.86	0.90
	OrgCommitment_5_1B	0.77	0.88	
	OrgCommitment_5_1C	0.71	0.89	
	OrgCommitment_5_1D	0.76	0.88	
	OrgCommitment_5_1E	0.74	0.89	
Quality improvement strategies	QI_5_2A	0.70	0.93	0.92
	QI_5_2B	0.82	0.89	
	QI_5_2C	0.90	0.86	
	QI_5_2D	0.84	0.88	

The CRCT items with *α* < 0.7, a low level of correlation (<0.4), and where removal of the item did not affect the total *α* (i.e., items which did not contribute meaningfully to the sub-component) were assessed by the Working Group members based on the internal consistency results and the theoretical concepts to ensure acceptability of the level of consistency and validity. Of the 68 items, only one was found to increase the reliability of the tool if removed. The Appointment and Scheduling sub-component had three items and removing the third item (*Is it routine practice for the diabetes community based activities and programs to be planned or scheduled ahead of time?*) improved the internal consistency of the sub-component (from 0.66 to 0.69). The two-item Patient Access sub-component was retained given that information related to patients’ barriers to access are an important aspect of chronic disease care. Two items in the Communication and Cooperation subcomponent were removed due to redundancy (*α* = 0.91), (*Is patient satisfaction with health center services systematically and routinely assessed?)* and (*Do community, social, education and other programs and organizations have a strong health orientation?*).

#### Convergent validity

**Fig. 1:**
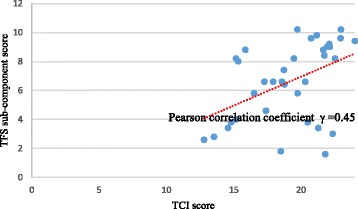
Scatter plot showing the moderate correlation between TCI and TSF sub-components of the CRCT

Thirty-five participants completed both scales. The TCI global score ranged from 12.8 to 24 while the TSF sub-component score ranges from 1.6 to 10.2. The TSF sub-component scale had a moderately significant positive correlation with the TCI, *r* = 0.45, *p* < 0.05 (Fig. 1).

## Discussion

It has been argued that complex health interventions need to be context-dependent to be most effective [12, 23, 40]. Within the context of Indigenous communities in Canada where the barriers encountered to providing optimal chronic care are numerous, sometimes unique and typically more exacerbated compared to their non-Indigenous counterparts, and resources are scarce [23, 40–42] the wisdom held by healthcare providers working in a community with regards to implementation of an intervention should not be underestimated. The concept of readiness has been consistently argued to be a key factor in influencing the success of health interventions [17, 18, 30, 33]. Trickett [43] argues that political, financial, educational, cultural, logistic, anthropological, and emotional barriers can affect even strongly supported interventions within a health system. The Canadian Indigenous context and the healthcare models that exist in First Nations communities became an important underpinning for the development of the FORGE AHEAD CRCT. The CRCT has been utilized within the FORGE AHEAD Program to assess readiness of healthcare teams providing type 2 diabetes care to Indigenous communities across the country. Furthermore, the CRCT is used within the program as the foundation to begin engagement and consultation discussions among healthcare team members who bring diverse backgrounds and perspectives with regards to the development of QI initiatives to enhance diabetes care in their communities.

According to Holt et al. [44] and Weiner et al. [18], researchers need to give greater attention to the development, testing, and refining of organizational readiness for change tools. The FORGE AHEAD CRCT was developed following a literature review that yielded the ABCD – SAT tool that was then adapted to the Canadian First Nations primary healthcare context using a consensus approach with thorough reviews and feedback from both First Nations community representatives and academic researchers. The CRCT pilot test results demonstrated good acceptability of the tool. Although length of the tool was noted as a drawback, all the questions were deemed important and necessary by respondents in understanding the current services available, and providing a comprehensive and complete picture of the existing healthcare delivery model in the community. Future work will aim to test the use of an on-line survey platform for communities with access to the internet. Such a platform may allow the information displayed to be customized for the respondent providing faster progression through the survey. Another possibility is having teams fill out the CRCT together rather than requesting individual team member completion first which could reduce the burden and focus teams together from the out-set. Concerns around the complexity of the language used were integrated into revisions following the pilot to simplify the language used in the tool.

Examination of the CRCT response data also demonstrated good acceptability of the tool with a high completion rate for all items except 8 that were left unanswered by more than 10% of the participants. Given the relatively high number of items in the CRCT, the proportion of unanswered items represents a small number. In addition, all items with higher proportions of missing data were those asking about the regional health planning and development, organizational commitment, or existing QI strategies at the health center. Given the fact that participants had a wide range of professional roles, this could be due to difficulty in understanding those questions and/or less knowledge about the issues that may have been more external to their role. In fact, of the four Health Directors/Program Managers who completed the CRCT, all four answered all the questions about regional health planning and development, organizational commitment and existing QI strategies. Reliability analysis demonstrated an acceptable *α* score for the 19 sub-components (ranging from 0.77 to 0.93), which demonstrated adequate internal consistency for standard scale development criteria. Based on a rigorous review guided by theoretical and statistical principles, only three out of 74 items were removed.

As expected, the TSF sub-component of the FORGE AHEAD CRCT showed evidence of convergent validity when compared to the validated TCI tool. This suggests that the sub-component of the CRCT intended to examine team functioning measures the same construct as the TCI. The moderate level of correlation is acceptable and can partly be explained by the fact that the TSF and TCI are structured differently with varying scales and types of questions. While the CRCT instrument has demonstrated validity and reliability, there are limitations. The small sample size did not allow for a confirmatory factory analysis to test whether the data collected from First Nations communities in Canada fits with the hypothesized measurement of readiness using the CRCT. Future confirmatory factor analysis studies with larger samples are suggested to better establish the validity of the FORGE AHEAD CRCT. Also, the psychometric properties of convergent validity for the entire CRCT could not be evaluated due to the non-existence of validated tools that measure a similar concept. As such, only the TSF sub-component was tested for convergent validity against the validated TCI.

The FORGE AHEAD Program is still in its implementation phase and as such, complete data are not yet available on the recruitment and retention of partnering communities, community and clinical team members QI initiatives, clinical outcomes, and cost analyses. Data on the use of the CRCT to facilitate QI initiatives and the impact of the CRCT on health-related outcomes will be available upon completion of the program. Detailed process evaluations including stakeholder interviews will inform further revisions to the CRCT as well as best practices for the successful implementation and scale-up of the tool. Process evaluations will also highlight how the CRCT enabled understanding and relationship building among healthcare team members working in Indigenous communities and between researchers and community stakeholders.

## Conclusions

In conclusion, the validated CRCT has been successfully used to support the implementation of the FORGE AHEAD Program and the health services changes that partnering First Nations communities have designed and undertaken to improve diabetes care.

## Additional files

Additional file 1: FORGE AHEAD: Clinical Readiness Consultation Tool – Community Feedback Form. The Community Feedback Form was used during the pilot of the CRCT and was includes a qualitative (open-ended) section for suggestions/comments and a five-point Likert scale to gather responses on the following: (1) appropriate language use for First Nations health clinics in Canada, (2) clarity of questions, (3) relevance of questions to health clinics in First Nations communities, (4) appropriate format for the tool, and, (5) helpfulness of examples provided for each question to complete the clinical assessment tool. (DOCX 43 kb)

Additional file 2: FORGE AHEAD: Clinical Readiness Consultation Tool. The 27-page tool has 4 main sections: 1) 1-page introduction describing the background, confidentiality, benefits, risks, reimbursement, consent, and contact information; 2) 1-page brief instruction (estimated time to complete, brief description of rating scales and how to submit the completed questionnaire); 3) general information (brief 8-item demographic profile); and 4) 5 main components and sub-components of healthcare systems important in chronic disease care. (DOCX 92 kb)

## Abbreviations

ABCD - SAT: Audit and Best Practice for Chronic Disease – Systems Assessment Tool
ACIC: Assessment of chronic illness care
CRCT: Clinical readiness consultation tool
FORGE AHEAD: Transformation of Indigenous Primary Healthcare Delivery
QI: Quality improvement
TCI: Team climate inventory
TSF: Team Structure and Function sub-component of the CRCT
