# Supplementary material for: The FORGE AHEAD clinical readiness consultation tool: a validated tool to assess clinical readiness for chronic disease care mobilization in Canada’s First Nations
Source: BMC Health Serv Res. 2017 Mar 23;17:233. doi: 10.1186/s12913-017-2175-6 (PMC5364708; doi:10.1186/s12913-017-2175-6)
Supplement: Supplementary file 1 — FORGE AHEAD: Clinical Readiness Consultation Tool – Community Feedback Form. The Community Feedback Form was used during the pilot of the CRCT and was includes a qualitative (open-ended) section for suggestions/comments and a five-point Likert scale to gather responses on the following: (1) appropriate language use for First Nations health clinics in Canada, (2) clarity of questions, (3) relevance of questions to health clinics in First Nations communities, (4) appropriate format for the tool, and, (5) helpfulness of examples provided for each question to complete the clinical assessment tool. (DOCX 43 kb) [file 12913_2017_2175_MOESM1_ESM.docx]

**FORGE AHEAD: Clinical Readiness Consultation Tool – Community Feedback Form**

Name: __________________________________________________________ Community: ______________________________________________________

The Forge Ahead clinical readiness assessment is an adapted version of a tool originally developed by a team in Australia. The tool has been used extensively in Indigenous communities and clinics in Australia for assessment of health clinics to support quality improvement initiatives to support the prevention and management of diabetes. This tool provides an opportunity to:

- **Look Back:** An assessment of your health clinic;
- **Look Forward:** Guidance on next steps in planning quality improvements; and
- **Assess progress:** An assessment of progress in achieving improvements at a clinic level.

Please review the cover letter and complete the Forge Ahead clinical readiness assessment. We would like an idea of how long it takes to complete – time yourself and enter the number of minutes it take on the following page. Once you have completed the assessment, provide your feedback below. Your perspective and feedback is important and will be considered when developing the final version of the tool to be used in Forge Ahead. Your individual answers are **confidential** and will not be shared.

| **Part 1: General Overview** | | | | | |
| --- | --- | --- | --- | --- | --- |
| **On a scale of 1 to 5, please indicate your level of agreement:**  (Answer each question clearly by placing an (⌧) in the box of your choice) | **Strongly Disagree**  **1** | **Disagree**  **2** | **Neutral**  **3** | **Agree**  **4** | **Strongly Agree**  **5** |
| The language is appropriate for use in First Nations health clinics in Canada |  |  |  |  |  |
| The questions in the survey are clearly written |  |  |  |  |  |
| The questions in the Clinical Readiness Assessment are relevant to health clinics in First Nations communities in Canada |  |  |  |  |  |
| The format of the Clinical Readiness Assessment is appropriate |  |  |  |  |  |
| The examples provided for each question help to complete the Clinical Readiness Assessment |  |  |  |  |  |

| **Part 2: Recommendations for improving the Clinical Readiness Assessment** |
| --- |
| How long did it take you to complete this assessment? _______________________ minutes |
| Please list any questions where the language is not appropriate for use in First Nations health clinics in Canada: |
| Please list any questions that are unclear. How would you recommend we change the questions to improve clarity? |
| Please list any questions that are not relevant for health clinics in First Nations communities in Canada: |
| Are there any questions that should be added? |
| Are there any questions that should be deleted? |
| Overall comments/suggestions: |
