# Supplementary material for: The FORGE AHEAD clinical readiness consultation tool: a validated tool to assess clinical readiness for chronic disease care mobilization in Canada’s First Nations
Source: BMC Health Serv Res. 2017 Mar 23;17:233. doi: 10.1186/s12913-017-2175-6 (PMC5364708; doi:10.1186/s12913-017-2175-6)
Supplement: Supplementary file 2 — FORGE AHEAD: Clinical Readiness Consultation Tool. The 27-page tool has 4 main sections: 1) 1-page introduction describing the background, confidentiality, benefits, risks, reimbursement, consent, and contact information; 2) 1-page brief instruction (estimated time to complete, brief description of rating scales and how to submit the completed questionnaire); 3) general information (brief 8-item demographic profile); and 4) 5 main components and sub-components of healthcare systems important in chronic disease care. (DOCX 92 kb) [file 12913_2017_2175_MOESM2_ESM.docx]

**FORGE AHEAD: Clinical Readiness Consultation Tool**

# INSTRUCTIONS

This questionnaire will take approximately 1 hour to complete. As you work your way through the questionnaire, read each question and circle the number you believe *best* reflects your views and practices at the health center. **There are no right or wrong answers.** The more honest and realistic your answers are, the better you can contribute to your community. A low response on the scale is not a negative reflection on the clinic – it is an assessment that will inform and guide your clinic through the clinical quality improvement initiatives. The scales provided on the questionnaire are divided into four categories:

1. Limited or no support (score 0-2)
2. Basic support (score 3-5)
3. Good support (score 6-8)
4. Fully developed support (score 9-11)

As you complete the assessment, we would like you to document your response on the scale and *why* you gave that response. Explaining your response is important so that the meaning of your response is clear when the results are reviewed at a later date.

**Where to send Your Completed Questionnaire?**

Please hand your completed questionnaire to your community facilitator.

| Name: |  |
| --- | --- |
| Fax #: |  |
| Email: |  |

**Thank you for your participation**

| GENERAL INFORMATION: | |
| --- | --- |
| 1. Are you: □ Male □ Female | |
| 1. Are you Indigenous? □ Yes □ No | |
| 1. Year of birth (yyyy): _____________ | |
| 1. How many years have you been working in Indigenous communities in Canada?   □ less than 12 months □ 1- 4 years □ 5 – 9 years □ 10 or more years □ Don’t know | |
| 1. How many years have you been working in this community?   □ less than 12 months □ 1- 4 years □ 5 – 9 years □ 10 or more years □ Don’t know | |
| 1. What is your current job title or role?   □ Family Physician  □ Health Director  □ Diabetes Nurse Educator  □ Administrative Assistant  □ Clerk  □ Clinic Director  □ Chart/Records Manager | □ Nurse Practitioner  □ Registered Nurse  □ Licensed Practical Nurse (LPN)  □ Community Health Representative  □ Pharmacist  □ Social Worker  □ Other (specify)_________________________ |
| 1. How many years of have you been practicing in your current role? _____________ | |
| 1. Number of ***half*** days per week you see patients: _____________ | |

| 1. **DELIVERY SYSTEM DESIGN**   *Effective delivery system of primary health care requires that service delivery infrastructure, staffing, and care processes (ex. annual foot examinations, HbA1c every three months, etc.) are designed to meet the specific needs of patients with diabetes, and their families. This involves more than simply adding additional interventions or programs to an existing system focused on acute care; it often necessitates significant changes to the organization of care.* | | | | | | | | | | | | |
| --- | --- | --- | --- | --- | --- | --- | --- | --- | --- | --- | --- | --- |
| - 1. **Team structure and function:** The following questions should be answered about the structure and function of your clinical ***team***. Every team looks different, so please keep your team in mind as you progress through the following 5 questions. For example, your team might consist of a physician and registered nurse, while a team in a different clinic might also have a pharmacist and physiotherapist. | | | | | | | | | | | | |
|  | ***Circle the number you believe best reflects your views and practices at the health center and explain why you gave that response in the justification section.*** | | | | | | | | | | | |
| **A) Are the practitioners required for a team approach to care available to your clinical team?**  For example: Is there an appropriate range of practitioners – medical, nursing, allied health, administrative, transport – always available to provide comprehensive, team-based, chronic illness (diabetes) care services? | No team approach; practitioners needed for team approach not available | | | Some efforts to establish a team approach; practitioners needed for team approach sometimes available, but not ongoing | | | Team approach becoming well established; practitioners needed for team approach usually available, becoming more secure and ongoing | | | Fully established team approach; secure, ongoing availability of practitioners needed for team approach | | |
|  | **0** | **1** | **2** | **3** | **4** | **5** | **6** | **7** | **8** | **9** | **10** | **11** |
|  | **Justification:** | | | | | | | | | | | |

| **B) Is the diabetes team leader defined and recognized? Does the leader have an appropriate level of formal authority within the practice team?**  For example: Does your team have a defined and recognized diabetes services coordinator to lead the chronic illness/diabetes care team (which is supported and back-filled when leave is taken)? If so, does this leader have the appropriate level of authority within the team to coordinate changes required to improve diabetes care at your clinic? | There is no team to lead; no team leader | | | Team leadership not clearly defined | | | Team leadership becoming defined and recognized, leader acquiring formal authority | | | Team leadership clearly defined and recognized, leader has formal authority | | |
| --- | --- | --- | --- | --- | --- | --- | --- | --- | --- | --- | --- | --- |
|  | **0** | **1** | **2** | **3** | **4** | **5** | **6** | **7** | **8** | **9** | **10** | **11** |
|  | **Justification:** | | | | | | | | | | | |
| **C) Are roles, responsibilities, and lines of reporting defined for all team members? Are these integrated into the delivery system?** | No team defined, or if there is a team, no definition of roles and responsibilities | | | Definition of team roles, lines of reporting and integration in system design are fair | | | Definition of team roles, lines of reporting and integration in system design are good | | | Definition of team roles, lines of reporting and integration in system design are very good | | |
|  | **0** | **1** | **2** | **3** | **4** | **5** | **6** | **7** | **8** | **9** | **10** | **11** |
|  | **Justification:** | | | | | | | | | | | |
| **D) Does communication and cohesion exist within the team? Does the team meet regularly? Are there established processes for effective decision making?**  For example: The team demonstrates that its members work well together to ensure all scheduled services and follow-up with patients with diabetes are carried out. | No team defined, or if there is a team, poor communication and cohesion | | | Fair communication and cohesion within the team; team meets irregularly (or not at all); decision-making is fair | | | Good communication and cohesion within the team; team meetings becoming regular; decision-making is good | | | Very good communication and cohesion within the team; team meetings regular; decision-making is very good | | |
|  | **0** | **1** | **2** | **3** | **4** | **5** | **6** | **7** | **8** | **9** | **10** | **11** |
|  | **Justification:** | | | | | | | | | | | |
| **E) Is there a strategic approach on your team to developing team members’ skills and roles?**  For example: Structured professional development plans and access to opportunities to develop skills and knowledge are in place to enhance roles and are supported by management. | No team defined, or if there is a team, development of team members’ skills and roles is poor | | | Development of team members’ skills and roles is fair | | | Development of team members’ skills and roles is good | | | Development of team members’ skills and roles is very good | | |
|  | **0** | **1** | **2** | **3** | **4** | **5** | **6** | **7** | **8** | **9** | **10** | **11** |
|  | **Justification:** | | | | | | | | | | | |

| - 1. **Clinical Leadership:** The following questions are designed to assess *clinical* leadership related to diabetes. For example, this could be a motivated healthcare worker dedicated to championing diabetes best practices in your clinic. | | | | | | | | | | | | |
| --- | --- | --- | --- | --- | --- | --- | --- | --- | --- | --- | --- | --- |
|  | ***Circle the number you believe best reflects your views and practices at the health center and explain why you gave that response in the justification section.*** | | | | | | | | | | | |
| **A) Is there a healthcare worker who provides leadership and direction clinically [related to diabetes]. Is it fully established and recognized in the area?**  For example: Is there a diabetes champion? | No or minimal clinical leadership | | | Clinical leadership emerging | | | Clinical leadership becoming established and recognized | | | Clinical leadership fully established and recognized | | |
|  | **0** | **1** | **2** | **3** | **4** | **5** | **6** | **7** | **8** | **9** | **10** | **11** |
|  | **Justification:** | | | | | | | | | | | |
| **B) Does clinical leadership contribute to the center’s vision for high quality care chronic illness/diabetes care for patients with diabetes?** | No vision for high quality care | | | Contribution of clinical leadership to center’s vision for high quality care is fair | | | Contribution of clinical leadership to center’s vision for high quality care is good | | | Contribution of clinical leadership to center’s vision for high quality care is very good | | |
|  | **0** | **1** | **2** | **3** | **4** | **5** | **6** | **7** | **8** | **9** | **10** | **11** |
|  | **Justification:** | | | | | | | | | | | |
| **C) Does clinical leadership help to ensure the center remains knowledgeable about research evidence? Is the evidence interpreted and appropriately applied to the center’s clinical services and programs?**  For example: Clinical leaders in diabetes care provide in-services to other team members when best practice guidelines are adjusted or new research becomes available. | No use of current research evidence | | | Contribution of clinical leadership to knowledge and application is fair | | | Contribution of clinical leadership to knowledge and application is good | | | Contribution of clinical leadership to knowledge and application is very good | | |
|  | **0** | **1** | **2** | **3** | **4** | **5** | **6** | **7** | **8** | **9** | **10** | **11** |
|  | **Justification:** | | | | | | | | | | | |

| - 1. **Appointments and Scheduling** | | | | | | | | | | | | |
| --- | --- | --- | --- | --- | --- | --- | --- | --- | --- | --- | --- | --- |
|  | ***Circle the number you believe best reflects your views and practices at the health center and explain why you gave that response in the justification section.*** | | | | | | | | | | | |
| **A) Is there an established appointment system for diabetes? Does it have the flexibility to systematically accommodate required needs ie. drop-ins, long or family consultations, patients seeing multiple providers in a single visit?** | No appointment system | | | Some appointments made; flexibility is ad hoc | | | Appointment system becoming established; flexibility becoming systematic | | | Appointment system fully established; flexibility is systematic | | |
|  | **0** | **1** | **2** | **3** | **4** | **5** | **6** | **7** | **8** | **9** | **10** | **11** |
|  | **Justification:** | | | | | | | | | | | |
| **B) Are there clinics/sessions with specialists (i.e. opthalmologists, foot care, nephrology, etc.) available for appointments in your clinic?** | Specific clinics and/or sessions not used | | | Specific clinics and/or sessions used in ad hoc way | | | Specific clinics and/or sessions becoming part of routine practice | | | Specific clinics and/or sessions part of routine practice | | |
|  | **0** | **1** | **2** | **3** | **4** | **5** | **6** | **7** | **8** | **9** | **10** | **11** |
|  | **Justification:** | | | | | | | | | | | |
| **C) Is it routine practice for the diabetes community based activities and programs to be planned or scheduled ahead of time?** | No or few community based activities | | | Scheduling of activities/programs is ad hoc | | | Planning/scheduling of activities/programs becoming routine practice | | | Planning/scheduling of activities/programs is routine practice | | |
|  | **0** | **1** | **2** | **3** | **4** | **5** | **6** | **7** | **8** | **9** | **10** | **11** |
|  | **Justification:** | | | | | | | | | | | |

| - 1. **Care Planning:** Please answer the following questions concerning structured, diabetes-specific care plans. A care plan may consist of patients/families health status, health care goals, and strategies (including self-management strategies) to improve diabetes management. | | | | | | | | | | | | |
| --- | --- | --- | --- | --- | --- | --- | --- | --- | --- | --- | --- | --- |
|  | ***Circle the number you believe best reflects your views and practices at the health center and explain why you gave that response in the justification section.*** | | | | | | | | | | | |
| **A) Are patient care plans a part of routine practice?**  For example: There is a structured chronic disease management care plan adapted to context, available and routinely utilized. | No or minimal care planning | | | Care planning is ad hoc | | | Care planning becoming part of routine practice | | | Care planning part of routine practice | | |
|  | **0** | **1** | **2** | **3** | **4** | **5** | **6** | **7** | **8** | **9** | **10** | **11** |
|  | **Justification:** | | | | | | | | | | | |
| **B) Is care planning consistent with best practice guidelines? Is care planning done jointly by providers and patients /families?**  For example: Care planning includes goal setting and the incorporation of self-management goals and strategies**.** | No or minimal care planning | | | Some elements included | | | Most elements included | | | All elements included | | |
|  | **0** | **1** | **2** | **3** | **4** | **5** | **6** | **7** | **8** | **9** | **10** | **11** |
|  | **Justification:** | | | | | | | | | | | |

| - 1. **Systematic approach to follow-up** | | | | | | | | | | | | |
| --- | --- | --- | --- | --- | --- | --- | --- | --- | --- | --- | --- | --- |
|  | ***Circle the number you believe best reflects your views and practices at the health center and explain why you gave that response in the justification section.*** | | | | | | | | | | | |
| **A) Do all patients receive regular follow-up in accordance with best practice?** | No or minimal follow-up of patients | | | Follow-up of patients for regular reviews is ad hoc | | | Follow-up of patients for regular reviews is becoming part of routine practice | | | Follow-up of patients for regular reviews is routine practice | | |
|  | **0** | **1** | **2** | **3** | **4** | **5** | **6** | **7** | **8** | **9** | **10** | **11** |
|  | **Justification:** | | | | | | | | | | | |
| **B) Is follow-up on abnormal pathology and other test results a systematic part of routine practice?**  For example: abnormal creatinine levels. | No or minimal processes for following up abnormal results | | | Follow-up of abnormal test results is ad hoc | | | Follow-up of abnormal test results is becoming part of routine practice | | | Follow-up of abnormal test results is routine practice | | |
|  | **0** | **1** | **2** | **3** | **4** | **5** | **6** | **7** | **8** | **9** | **10** | **11** |
|  | **Justification:** | | | | | | | | | | | |
| **C) Does health centre staff utilize available resources to enhance follow-up?**  For example: Do health center staff use taxis to assist patients to get to the clinic? Do staff use local radio/newspaper advertising to remind patients to follow-up at the clinic? | No or minimal use of available resources to enhance follow-up | | | Use of available resources to enhance follow-up is fair | | | Use of available resources to enhance follow-up is good | | | Use of available resources to enhance follow-up is very good | | |
|  | **0** | **1** | **2** | **3** | **4** | **5** | **6** | **7** | **8** | **9** | **10** | **11** |
|  | **Justification:** | | | | | | | | | | | |
| **D) Are electronic or other types of flags and reminders used to support patient care in diabetes?**  For example: Flags or reminders are used when an HbA1c test is due. | No electronic flags/ reminders | | | Flags/reminders sometimes used to support patient care | | | Flags/reminders usually used to support patient care | | | Flags/reminders consistently used to support patient care | | |
|  | **0** | **1** | **2** | **3** | **4** | **5** | **6** | **7** | **8** | **9** | **10** | **11** |
|  | **Justification:** | | | | | | | | | | | |

| - 1. **Continuity of Care** | | | | | | | | | | | | | | | | | | |
| --- | --- | --- | --- | --- | --- | --- | --- | --- | --- | --- | --- | --- | --- | --- | --- | --- | --- | --- |
|  | | ***Circle the number you believe best reflects your views and practices at the health center and explain why you gave that response in the justification section.*** | | | | | | | | | | | | | | | | |
| **A) Is the delivery system designed to enhance continuity of care for patients with diabetes by having the following elements: scheduled follow-up visits, continuity of provider(s), case management, shared patient records?** | | Delivery system is not designed to enhance continuity of care | | | | | | Delivery system beginning to be designed to enhance continuity of care (some elements in place) | | | | | Delivery system quite well designed to enhance continuity of care (most elements in place) | | | Delivery system very well designed to enhance  continuity of care (all or almost all elements in place) | | |
|  |  | **0** | | **1** | | **2** | | **3** | | **4** | | **5** | **6** | **7** | **8** | **9** | **10** | **11** |
|  |  | **Justification:** | | | | | | | | | | | | | | | | |
| **B) Does the delivery system between hospital(s) and health centre effectively following the discharge of patients with diabetes?**  For example: Follow-up arrangement/timely discharge summaries. | | No or minimal communication between hospital and the health center post-discharge | | | | | | Post-discharge communication between hospital and the health center is on an ad hoc basis only | | | | | System for routine post- discharge communication between hospital and the health center becoming established | | | System for routine post- discharge communication between hospital and the health center fully established | | |
|  |  | **0** | | **1** | | **2** | | **3** | | **4** | | **5** | **6** | **7** | **8** | **9** | **10** | **11** |
|  |  | **Justification:** | | | | | | | | | | | | | | | | |
| - 1. **Patient Access** | | | | | | | | | | | | | | | | | | |
|  | | ***Circle the number you believe best reflects your views and practices at the health center and explain why you gave that response in the justification section.*** | | | | | | | | | | | | | | | | |
| **A) Do health center design and processes address physical/transport barriers to access for patients with diabetes?**  For example: transport support for referrals (as required)? | | No or minimal attention given to barriers | | | | | | Barriers beginning to be addressed but many remain | | | | | Barriers addressed quite well but some remain | | | Barriers addressed very well and few or none remain | | |
|  |  | **0** | | **1** | | **2** | | **3** | | **4** | | **5** | **6** | **7** | **8** | **9** | **10** | **11** |
|  |  | **Justification:** | | | | | | | | | | | | | | | | |
| **B) Do health center design and processes address patient barriers to access related to communication?**  For example: the use of translators (as required)? | | No or minimal attention given to barriers | | | | | | Barriers beginning to be addressed but many remain | | | | | Barriers addressed quite well but some remain | | | Barriers addressed very well and few or none remain | | |
|  |  | **0** | | **1** | | **2** | | **3** | | **4** | | **5** | **6** | **7** | **8** | **9** | **10** | **11** |
|  |  | **Justification:** | | | | | | | | | | | | | | | | |
| - 1. **Cultural Competence / Knowledge** | | | | | | | | | | | | | | | | | | |
|  | ***Circle the number you believe best reflects your views and practices at the health center and explain why you gave that response in the justification section.*** | | | | | | | | | | | | | | | | | |
| **A) Is there a systematic approach to ensuring that all health center staff providing care are culturally competent and knowledgeable through staff orientation and training**? | No or minimal attention given to cultural competence; not included in orientation and training | | | | | | Level of attention to cultural competence is fair; sometimes included in orientation and training | | | | | | Level of attention to cultural competence is good; usually included in orientation and training | | | Level of attention to cultural competence is very good; always included in orientation and training | | |
|  | **0** | | **1** | | **2** | | **3** | | **4** | | **5** | | **6** | **7** | **8** | **9** | **10** | **11** |
|  | **Justification:** | | | | | | | | | | | | | | | | | |
| **B) Is cultural competence incorporated into the mission/ vision statement of the health centre?** | Cultural competence is not incorporated into the mission/vision statement | | | | | | Cultural competence is incorporated into the mission/vision statement; this statement is not adhered to | | | | | | Cultural competence is incorporated into the mission/vision statement; this statement is sometimes adhered to | | | Cultural competence is incorporated into the mission/vision statement; this statement is always adhered to | | |
|  | **0** | | **1** | | **2** | | **3** | | **4** | | **5** | | **6** | **7** | **8** | **9** | **10** | **11** |
|  | **Justification:** | | | | | | | | | | | | | | | | | |
| **C) Is there a systematic approach to ensuring that all health staff and visiting professionals and supports are aware of the significance of cultural considerations and competence in practice?**  For example: through orientation and training? | No or minimal attention given to cultural competence; not included in orientation and training | | | | | | Level of attention to cultural competence is fair; sometimes included in orientation and training | | | | | | Level of attention to cultural competence is good; usually included in orientation and training | | | Level of attention to cultural competence is very good; always included in orientation and training | | |
|  | **0** | | **1** | | **2** | | **3** | | **4** | | **5** | | **6** | **7** | **8** | **9** | **10** | **11** |
|  | **Justification:** | | | | | | | | | | | | | | | | | |
| **D) Is Indigenous knowledge and experience respected?**  For example: Inclusion of all Indigenous team members (including administration, community liaison, outreach officers, community transport officers) in team planning processes to ensure comprehensive input to chronic disease management. | No or minimal respect for Indigenous knowledge or experience | | | | | | Respect for Indigenous knowledge and experience is fair | | | | | | Respect for Indigenous knowledge and experience is good | | | Respect for Indigenous knowledge and experience is very good | | |
|  | **0** | | **1** | | **2** | | **3** | | **4** | | **5** | | **6** | **7** | **8** | **9** | **10** | **11** |
|  | **Justification:** | | | | | | | | | | | | | | | | | |

| - 1. **Physical Infrastructure** | | | | | | | | | | | | |
| --- | --- | --- | --- | --- | --- | --- | --- | --- | --- | --- | --- | --- |
|  | ***Circle the number you believe best reflects your views and practices at the health center and explain why you gave that response in the justification section.*** | | | | | | | | | | | |
| **A) Is the physical infrastructure of the health centre suitable for the provision of care?**  For example: enough office space for staff and clinicians, adequate lodging for staff and clinicians, availability of examination rooms? | Physical infrastructure unsuitable | | | Physical infrastructure somewhat suitable | | | Physical infrastructure quite suitable | | | Physical infrastructure highly suitable | | |
|  | **0** | **1** | **2** | **3** | **4** | **5** | **6** | **7** | **8** | **9** | **10** | **11** |
|  | **Justification:** | | | | | | | | | | | |
| **B) Are supplies appropriate and available?**  For example: Glucose test strips, insulin needles, syringes, tape measures | Appropriateness and availability of consumables is poor | | | Appropriateness and availability of consumables is fair | | | Appropriateness and availability of consumables is good | | | Appropriateness and availability of consumables is very good | | |
|  | **0** | **1** | **2** | **3** | **4** | **5** | **6** | **7** | **8** | **9** | **10** | **11** |
|  | **Justification:** | | | | | | | | | | | |
| **C) Is the equipment in the health centre appropriate and available?** Is **it good quality and well maintained (for example, the equipment does not need to be shared between rooms, or borrowed from other consulting areas due to limited availability)?**  For example: Functioning/calibrated scales, blood pressure equipment. | Equipment appropriateness, quality and maintenance is poor | | | Equipment appropriateness, quality and maintenance are fair | | | Equipment appropriateness, quality and maintenance are good | | | Equipment appropriateness, quality and maintenance are very good | | |
|  | **0** | **1** | **2** | **3** | **4** | **5** | **6** | **7** | **8** | **9** | **10** | **11** |
|  | **Justification:** | | | | | | | | | | | |

| 1. **Information Systems and Decision Support**   *Effective health centers ensure that paper or electronic information systems contain up-to-date patient information that is used to support the planning and delivery of care, including decision support. Evidence-based guidelines and other resources should be available through the systems in formats that are appropriate and accessible for all members of the health care team. In addition, advice may be available through specialist collaborations and other mechanisms.* | | | | | | | | | | | | |
| --- | --- | --- | --- | --- | --- | --- | --- | --- | --- | --- | --- | --- |
| - 1. **Maintenance and use of an electronic or paper diabetes registry** | | | | | | | | | | | | |
|  | ***Circle the number you believe best reflects your views and practices at the health center and explain why you gave that response in the justification section.*** | | | | | | | | | | | |
| **A) Is the diabetes registry regularly reviewed according to an established protocol? Is it up to date, including record of place of residence and health insurance number?**  For example: A list of all patients with diabetes in the service population is maintained and up-to-date/ | No registry | | | List available but not reviewed and out of date (covers less than 80% of patients, up-to-date residence and insurance information sometimes recorded) | | | List available, irregularly reviewed and reasonably up to date (covers 80% or more of patients, up-to-date residence and insurance information usually recorded) | | | List available, regularly reviewed and up to date (covers all patients, up-to-date residence and insurance information always recorded) | | |
|  | **0** | **1** | **2** | **3** | **4** | **5** | **6** | **7** | **8** | **9** | **10** | **11** |
|  | **Justification:** | | | | | | | | | | | |
| **B) Do you use a diabetes registry as part of routine practice?** | No registry | | | Implementation of strategies to reach patient groups is ad hoc | | | Implementation of strategies to reach patient groups becoming routine practice | | | Implementation of strategies to reach patient groups is routine practice | | |
|  | **0** | **1** | **2** | **3** | **4** | **5** | **6** | **7** | **8** | **9** | **10** | **11** |
|  | **Justification:** | | | | | | | | | | | |

| - 1. **Evidence-based guidelines for diabetes** | | | | | | | | | | | | |
| --- | --- | --- | --- | --- | --- | --- | --- | --- | --- | --- | --- | --- |
|  | ***Circle the number you believe best reflects your views and practices at the health center and explain why you gave that response in the justification section.*** | | | | | | | | | | | |
| **A) Are evidence-based guidelines for diabetes available and suitable to your community?**  For example: CDA clinical practice/care guidelines, Canadian Hypertensive Education Guidelines. | No or minimal availability or accessibility of evidence-based resources | | | Availability and accessibility of evidence-based resources is fair | | | Availability and accessibility of evidence-based resources is good | | | Availability and accessibility of evidence-based resources is very good | | |
|  | **0** | **1** | **2** | **3** | **4** | **5** | **6** | **7** | **8** | **9** | **10** | **11** |
|  | **Justification:** | | | | | | | | | | | |
| **B) Are clinical practice guidelines for diabetes used as part of routine practice?** | No or minimal use of evidence- based resources | | | Use of evidence-based resources is ad hoc | | | Use of evidence-based resources becoming part of routine practice | | | Use of evidence-based resources is part of routine practice | | |
|  | **0** | **1** | **2** | **3** | **4** | **5** | **6** | **7** | **8** | **9** | **10** | **11** |
|  | **Justification:** | | | | | | | | | | | |
| **C) Are staff trained and oriented to new guidelines applicable to patients with diabetes?**  For example: staff in-service training when new guidelines are published? | No or minimal staff training in use of evidence-based resources | | | Staff training in use of evidence-based resources is fair | | | Staff training in use of evidence-based resources is good | | | Staff training in use of evidence-based resources is very good | | |
|  | **0** | **1** | **2** | **3** | **4** | **5** | **6** | **7** | **8** | **9** | **10** | **11** |
|  | **Justification:** | | | | | | | | | | | |

| - 1. **Specialist and generalist collaboration** | | | | | | | | | | | | |
| --- | --- | --- | --- | --- | --- | --- | --- | --- | --- | --- | --- | --- |
|  | ***Circle the number you believe best reflects your views and practices at the health center and explain why you gave that response in the justification section.*** | | | | | | | | | | | |
| **A) Is there a strategic approach that results in established processes and mechanism of communication and collaboration between specialists and generalists?**  For example, health center staff and clinicians communicate and collaborate with specialists about patient needs and care? | No or minimal specialist- generalist collaboration – i.e. traditional referral only, specialist referral only | | | Specialist-generalist collaboration is fair | | | Specialist-generalist collaboration is good | | | Specialist-generalist collaboration is very good | | |
|  | **0** | **1** | **2** | **3** | **4** | **5** | **6** | **7** | **8** | **9** | **10** | **11** |
|  | **Justification:** | | | | | | | | | | | |

| 1. **Component 3 – Self-management support**   *Self-management support refers to health center structures and processes that support patients and their families to play a major role in maintaining their health, managing their health, and achieving safe and healthy environments. Effective self-management support strategies include assessing and documenting self-management needs and activities, providing education and support and behavior change interventions, and promoting peer support.* | | | | | | | | | | | | | | | | | | | | |
| --- | --- | --- | --- | --- | --- | --- | --- | --- | --- | --- | --- | --- | --- | --- | --- | --- | --- | --- | --- | --- |
| - 1. **Self-management support, assessment and documentation** | | | | | | | | | | | | | | | | | | | | |
|  | ***Circle the number you believe best reflects your views and practices at the health center and explain why you gave that response in the justification section.*** | | | | | | | | | | | | | | | | | | | |
| **A) Is self-management for patients with diabetes a central, strategic part of routine diabetes care?**  For example: The patient’s role in maintaining their health, managing their health problems, and promoting a safe and healthy environment, is a central tenet of self-management support and routinely discussed during patient visits. The goal is for patients to be actively engaged in the management of their diabetes. | No or minimal support for self- management. | | | | | | Fair support for self- management. Self-management is part of ad hoc care. | | | | | | Good support for self- management. Self-management is becoming part of routine care. | | | | | Very good support for self- management. Self-management is routinely part of care. | | |
|  | **0** | | **1** | | **2** | | **3** | | **4** | | **5** | | **6** | | **7** | | **8** | **9** | **10** | **11** |
|  | **Justification:** | | | | | | | | | | | | | | | | | | | |
| **B) Is self-management by patients with diabetes routinely assessed and documented in a standardized way?**  For example: Assessment, goal-setting, action planning, problem solving, and follow-up for self-management support is routinely done and documented on standardized forms. | Self-management needs are rarely assessed | | | | | | Self-management needs sometimes assessed and documented but on an ad hoc basis only | | | | | | Assessment and documentation of self-management needs becoming routine practice | | | | | Assessment and documentation of self- management needs is routine practice | | |
|  | **0** | | **1** | | **2** | | **3** | | **4** | | **5** | | **6** | | **7** | | **8** | **9** | **10** | **11** |
|  | **Justification:** | | | | | | | | | | | | | | | | | | | |
| **C) Does the health centre utilize tools designed to assist patients to adhere to self-management programs, set goals, track their progress, and to understand the reasons for health visits?**  For example: a patient held record could be a diary to record blood sugar, insulin dose – the patient would then use this information to adjust their therapy. | No or minimal use of patient held records | | | | | | Use of patient held records is ad hoc | | | | | | Use of patient held records becoming part of routine practice | | | | | Use of patient held records is part of routine practice | | |
|  | **0** | | **1** | | **2** | | **3** | | **4** | | **5** | | **6** | | **7** | | **8** | **9** | **10** | **11** |
|  | **Justification:** | | | | | | | | | | | | | | | | | | | |
| - 1. **Self-management education, behavioural risk reduction and peer support** | | | | | | | | | | | | | | | | | | | | |
|  | ***Circle the number you believe best reflects your views and practices at the health center and explain why you gave that response in the justification section.*** | | | | | | | | | | | | | | | | | | | |
| **A) Is diabetes self-management education routinely provided by staff with *recognized training and skills in self- management*?** | No or minimal self- management education or support | | | | | | Some self-management education and support by staff with limited training and skills | | | | | | Good self-management education and support by staff with relevant training and skills | | | | | Very good self-management education and support by staff with relevant training and skills | | |
|  | **0** | | **1** | | **2** | | **3** | | **4** | | **5** | | **6** | | **7** | | **8** | **9** | **10** | **11** |
|  | **Justification:** | | | | | | | | | | | | | | | | | | | |
| **B) Are families involved in self-management education and support activities as part of routine practice?**  For example: Consider patient behavior related to smoking, nutrition and physical activity, and emotional/economic factors that may influence these behaviours. Does clinic staff make efforts to address concerns of patients and families, with mechanisms for families to be involved in support or programs? | | No or minimal engagement of families in education/support activities | | | | | | Engagement of families in education/ support activities but on an ad hoc basis only | | | | | | Engagement of families in education/ support activities becoming routine practice | | | | Engagement of families in education/ support activities is routine practice | | |
|  |  | **0** | | **1** | | **2** | | **3** | | **4** | | **5** | | **6** | | **7** | **8** | **9** | **10** | **11** |
|  |  | **Justification:** | | | | | | | | | | | | | | | | | | |
| **C) Is there a systematic approach to behaviour change interventions?**  For example: promotion of positive lifestyle changes and interventions?  **Are brief interventions routinely provided by staff with training and skills in behavioural intervention?**  For example: Motivational interviewing techniques are known and utilized to maximize opportunities for patients to change behaviours. | | No or minimal provision of behaviour change interventions | | | | | | Some behavioural interventions provided but by staff with limited relevant training and skills | | | | | | Behavioural interventions by staff with relevant training and skills becoming part of routine practice | | | | Behavioural interventions by staff with relevant training and skills part of routine practice | | |
|  |  | **0** | | **1** | | **2** | | **3** | | **4** | | **5** | | **6** | | **7** | **8** | **9** | **10** | **11** |
|  |  | **Justification:** | | | | | | | | | | | | | | | | | | |
| **D) Are good quality culturally relevant educational resources (ie. nutritional guides and meal planning resources, smoking cessation resources, etc.) used for patients and families to support behavioural risk reduction self-management? Is this part of routine practice?** | | No or minimal use of resources to support self- management | | | | | | Some use of resources to support self-management | | | | | | Use of resources to support self-management becoming routine practice | | | | Use of resources to support self-management is routine practice | | |
|  |  | **0** | | **1** | | **2** | | **3** | | **4** | | **5** | | **6** | | **7** | **8** | **9** | **10** | **11** |
|  |  | **Justification:** | | | | | | | | | | | | | | | | | | |

| 1. **Component 4 – Linkages with Community Resources and Other Health Services**   *Good links and partnerships between the health center and the community, and other community based organizations and programs are important in primary health care for patients with diabetes. They allow the center to have effective community input to planning, to link patients to outside resources, to work with population groups out in the community and to contribute to regional activities such as service planning and the development of resources.* | | | | | | | | | | | | |
| --- | --- | --- | --- | --- | --- | --- | --- | --- | --- | --- | --- | --- |
| - 1. **Communication and cooperation of the health center and other community based organizations and programs** | | | | | | | | | | | | |
|  | ***Circle the number you believe best reflects your views and practices at the health center and explain why you gave that response in the justification section.*** | | | | | | | | | | | |
| **A) Is there community input as to how the health center is run?** | No community input | | | Community input is fair | | | Community input is good | | | Community input is very good | | |
|  | **0** | **1** | **2** | **3** | **4** | **5** | **6** | **7** | **8** | **9** | **10** | **11** |
|  | **Justification:** | | | | | | | | | | | |
| **B) Does the community recognize the value of having a team of diabetes professionals in the community to provide care for patients with diabetes?** | No community recognition | | | Community recognition is fair | | | Community recognition is good | | | Community recognition is very good | | |
|  | **0** | **1** | **2** | **3** | **4** | **5** | **6** | **7** | **8** | **9** | **10** | **11** |
|  | **Justification:** | | | | | | | | | | | |

| **C) Are patients with diabetes involved in planning how the health center is run, and encouraged to provide feedback?** | No service population involvement in planning and feedback | | | Service population involvement in planning and feedback is ad hoc. | | | Service population involvement in planning and feedback is becoming systematic | | | Service population involvement in planning and feedback is systematic | | |
| --- | --- | --- | --- | --- | --- | --- | --- | --- | --- | --- | --- | --- |
|  | **0** | **1** | **2** | **3** | **4** | **5** | **6** | **7** | **8** | **9** | **10** | **11** |
|  | **Justification:** | | | | | | | | | | | |
| **D) Is patient satisfaction with health center services systematically and routinely assessed?** | Client satisfaction never or rarely assessed | | | Assessment of client satisfaction is ad hoc | | | Assessment of client satisfaction is becoming systematic and routine | | | Assessment of client satisfaction is systematic and routine | | |
|  | **0** | **1** | **2** | **3** | **4** | **5** | **6** | **7** | **8** | **9** | **10** | **11** |
|  | **Justification:** | | | | | | | | | | | |
| **E) Are there well-functioning arrangements with relevant community groups for the health center to work with?** | No or poor partnerships with community groups | | | Partnerships with community groups are fair | | | Partnerships with community groups are good | | | Partnerships with community groups are very good | | |
|  | **0** | **1** | **2** | **3** | **4** | **5** | **6** | **7** | **8** | **9** | **10** | **11** |
|  | **Justification:** | | | | | | | | | | | |
| **F) Do community, social, education and other programs and organizations have a strong health orientation?**  For example: healthy food at council meetings, smoking policies. | Health orientation of community programs is weak | | | Health orientation of community programs is fair | | | Health orientation of community programs is good | | | Health orientation of community programs is very good | | |
|  | **0** | **1** | **2** | **3** | **4** | **5** | **6** | **7** | **8** | **9** | **10** | **11** |
|  | **Justification:** | | | | | | | | | | | |

| - 1. **Linking health center patients to community resources** | | | | | | | | | | | | |
| --- | --- | --- | --- | --- | --- | --- | --- | --- | --- | --- | --- | --- |
|  | ***Circle the number you believe best reflects your views and practices at the health center and explain why you gave that response in the justification section.*** | | | | | | | | | | | |
| **A) Are there systematic arrangements in place to link individual clients with diabetes to community health and health-related resources?**  For example: Consider team member’s knowledge about what is available in the community and whether patients with diabetes are referred to these groups? | No or minimal arrangements for linking clients to community resources | | | Arrangements for linking clients to community resources ad hoc | | | Arrangements for linking clients to community resources becoming systematic | | | Arrangements for linking clients to community resources are systematic | | |
|  | **0** | **1** | **2** | **3** | **4** | **5** | **6** | **7** | **8** | **9** | **10** | **11** |
|  | **Justification:** | | | | | | | | | | | |
| **B) Is the community resource directory that supports systematic arrangements comprehensive, regularly updated, easily accessible and widely used by staff?** | No resource directory | | | Resource directory – comprehensiveness, updating accessibility and use are marginal | | | Resource directory – comprehensiveness, updating accessibility and use are good | | | Resource directory – comprehensiveness, updating accessibility and use are excellent | | |
|  | **0** | **1** | **2** | **3** | **4** | **5** | **6** | **7** | **8** | **9** | **10** | **11** |
|  | **Justification:** | | | | | | | | | | | |
| **C) Are linkage arrangements relating to these resources well integrated into staff orientation and in-service training programs?** | No or minimal integration of linkage arrangements in staff orientation or training | | | Integration of linkage arrangements in staff orientation or training is fair | | | Integration of linkage arrangements in staff orientation or training is good | | | Integration of linkage arrangements in staff orientation or training is very good | | |
|  | **0** | **1** | **2** | **3** | **4** | **5** | **6** | **7** | **8** | **9** | **10** | **11** |
|  | **Justification:** | | | | | | | | | | | |

| - 1. **Community outreach** | | | | | | | | | | | | |
| --- | --- | --- | --- | --- | --- | --- | --- | --- | --- | --- | --- | --- |
|  | ***Circle the number you believe best reflects your views and practices at the health center and explain why you gave that response in the justification section.*** | | | | | | | | | | | |
| **A) Are staff engaged in community health promotion/development activities?**  For example: In pre-schools and schools; men, women and youth groups; community centers; community stores. | No or minimal staff engagement in community health promotion/ development | | | Level of staff engagement in community health promotion/development is fair | | | Level of staff engagement in community health promotion/development is good | | | Level of staff engagement in community health promotion/development is very good | | |
|  | **0** | **1** | **2** | **3** | **4** | **5** | **6** | **7** | **8** | **9** | **10** | **11** |
|  | **Justification:** | | | | | | | | | | | |
| **B) Are community activities well-designed to meet the identified needs of patients with diabetes?**  For example, community activities recognize and make adjustments for patients with mobility or vision problems? | Marginal design of community activities | | | Design of community activities is fair | | | Design of community activities is good | | | Design of community activities is very good | | |
|  | **0** | **1** | **2** | **3** | **4** | **5** | **6** | **7** | **8** | **9** | **10** | **11** |
|  | **Justification:** | | | | | | | | | | | |
| **C) Are health center activities integrated within the community?** | Marginal integration of community activities into center’s programs. | | | Integration of community activities into center’s programs is fair | | | Integration of community activities into center’s programs is good | | | Integration of community activities into center’s programs is very good | | |
|  | **0** | **1** | **2** | **3** | **4** | **5** | **6** | **7** | **8** | **9** | **10** | **11** |
|  | **Justification:** | | | | | | | | | | | |

| - 1. **Regional health planning and development of health resources** | | | | | | | | | | | | |
| --- | --- | --- | --- | --- | --- | --- | --- | --- | --- | --- | --- | --- |
|  | ***Circle the number you believe best reflects your views and practices at the health center and explain why you gave that response in the justification section.*** | | | | | | | | | | | |
| **A) Are health center staff actively engaged in and promote regional planning?**  For example: Consider whether there are opportunities for team members to attend regional meetings and present local data related to chronic illness and diabetes care and management with relevant feedback to the health center team. | No or minimal engagement in regional planning | | | Level of engagement in regional planning is fair | | | Level of engagement in regional planning is good | | | Level of engagement in regional planning is very good | | |
|  | **0** | **1** | **2** | **3** | **4** | **5** | **6** | **7** | **8** | **9** | **10** | **11** |
|  | **Justification:** | | | | | | | | | | | |
| **B) Do health center staff actively contribute to the development and promotion of standard resources for health services that have region-wide relevance in diabetes?** | No or minimal contribution to the development of resources | | | Contribution to the development of resources is fair | | | Contribution to the development of resources is good | | | Contribution to the development of resources is very good | | |
|  | **0** | **1** | **2** | **3** | **4** | **5** | **6** | **7** | **8** | **9** | **10** | **11** |
|  | **Justification:** | | | | | | | | | | | |
| **C) Are local plans systematically used in regional planning processes and allocation of resources?** | No or minimal use of community plans | | | Use of community plans is ad hoc | | | Use of community plans is becoming systematic | | | Use of community plans is systematic. | | |
|  | **0** | **1** | **2** | **3** | **4** | **5** | **6** | **7** | **8** | **9** | **10** | **11** |
|  | **Justification:** | | | | | | | | | | | |

| 1. **Component 5 – Local health center organizational influence and integration**   *Primary health care for patients with diabetes will be more effective if there is an organizational culture that is committed to addressing the needs of patients, promotes good relationships and safe communication, and promotes high quality care and quality improvement. In addition, effective primary healthcare requires the integration of the health center’s system components.* | | | | | | | | | | | | |
| --- | --- | --- | --- | --- | --- | --- | --- | --- | --- | --- | --- | --- |
| - 1. **Organizational commitment** | | | | | | | | | | | | |
|  | ***Circle the number you believe best reflects your views and practices at the health center and explain why you gave that response in the justification section.*** | | | | | | | | | | | |
| **A) Does the local health center strategic plan reflect commitment to patients with diabetes?**  For example: vision statement, policies, financing, staffing? | No plans; little or no interest in a plan | | | Plans in place; level of commitment is fair | | | Plans in place; level of commitment is good | | | Plans in place; level of commitment is very good | | |
|  | **0** | **1** | **2** | **3** | **4** | **5** | **6** | **7** | **8** | **9** | **10** | **11** |
|  | **Justification:** | | | | | | | | | | | |
| **B) Is there specific funding for diabetes that is at an adequate level and long- term?** | No specific funding | | | Specific funding, level is fair and/or short term | | | Specific funding, level is good and/or medium term | | | Specific funding, level is very good and/or long term | | |
|  | **0** | **1** | **2** | **3** | **4** | **5** | **6** | **7** | **8** | **9** | **10** | **11** |
|  | **Justification:** | | | | | | | | | | | |

| **C) Do staffing levels meet the established need?**  **Are all the relevant roles defined and these roles reflected in job descriptions?** | Minimal staffing; no specific roles | | | Level of staffing is fair; some roles defined | | | Level of staffing is good; most roles defined and reflected in job descriptions | | | Level of staffing is very good; all roles defined and reflected in job descriptions | | |
| --- | --- | --- | --- | --- | --- | --- | --- | --- | --- | --- | --- | --- |
|  | **0** | **1** | **2** | **3** | **4** | **5** | **6** | **7** | **8** | **9** | **10** | **11** |
|  | **Justification:** | | | | | | | | | | | |
| **D) Are there good relationships and regular, clear communication among staff? Is morale high?** | Poor relationships and little or no communication. Morale is low | | | Relationships and communication are fair. Morale is fair | | | Relationships and communication are good. Morale is good | | | Relationships and communication are very good. Morale is very good | | |
|  | **0** | **1** | **2** | **3** | **4** | **5** | **6** | **7** | **8** | **9** | **10** | **11** |
|  | **Justification:** | | | | | | | | | | | |
| **E) Are there training and in-service opportunities for staff working in diabetes?** | Range of training and in- service opportunities is poor | | | Range of training and in-service opportunities is fair | | | Range of training and in-service opportunities is good | | | Range of training and in- service opportunities is very good | | |
|  | **0** | **1** | **2** | **3** | **4** | **5** | **6** | **7** | **8** | **9** | **10** | **11** |
|  | **Justification:** | | | | | | | | | | | |

| - 1. **Quality improvement strategies** | | | | | | | | | | | | |
| --- | --- | --- | --- | --- | --- | --- | --- | --- | --- | --- | --- | --- |
|  | ***Circle the number you believe best reflects your views and practices at the health center and explain why you gave that response in the justification section.*** | | | | | | | | | | | |
| **A) Does management/senior staff support quality improvement strategies for diabetes care?** | No or minimal senior staff support for quality improvement | | | Limited senior staff support for quality improvement | | | Senior staff support quality improvement but not fully or consistently | | | Quality improvement fully and consistently supported by senior staff | | |
|  | **0** | **1** | **2** | **3** | **4** | **5** | **6** | **7** | **8** | **9** | **10** | **11** |
|  | **Justification:** | | | | | | | | | | | |
| **B) Are systematic processes in place and used consistently?**  For example: Reoccurring assessment of health center performance using local data to review performance and plan improvements. | No or minimal quality improvement processes | | | Ad hoc quality improvement processes | | | Systematic quality improvement processes but not used consistently | | | Systematic quality improvement processes used consistently | | |
|  | **0** | **1** | **2** | **3** | **4** | **5** | **6** | **7** | **8** | **9** | **10** | **11** |
|  | **Justification:** | | | | | | | | | | | |
| **C) Is the diabetes client information system routinely used for quality improvement?** | The client information system is not used for quality improvement | | | Use of the system for reporting on center performance and quality improvement is ad hoc | | | Use of the system for reporting on center performance and quality improvement is becoming routine | | | Use of the system for reporting on center performance and quality improvement is routine | | |
|  | **0** | **1** | **2** | **3** | **4** | **5** | **6** | **7** | **8** | **9** | **10** | **11** |
|  | **Justification:** | | | | | | | | | | | |
| **D) Are systematic processes in place for dealing with errors or problems with diabetes care delivery? Do they include routine identification, examination of root causes and follow through appropriate action and regular review?** | No or minimal processes for dealing with errors or problems | | | Processes for dealing with errors or problems are ad hoc | | | Processes for dealing with errors becoming systematic | | | Processes for dealing with errors systematic | | |
|  | **0** | **1** | **2** | **3** | **4** | **5** | **6** | **7** | **8** | **9** | **10** | **11** |
|  | **Justification:** | | | | | | | | | | | |

| - 1. **Integration of health system components to achieve high quality care for patients with diabetes** | | | | | | | | | | | | |
| --- | --- | --- | --- | --- | --- | --- | --- | --- | --- | --- | --- | --- |
|  | ***Circle the number you believe best reflects your views and practices at the health center and explain why you gave that response in the justification section.*** | | | | | | | | | | | |
| **A) What is the level of integration across the health center?**  For example:   - How well the information system supports clinical decision making and self-management - How well the funding and human resources arrangements support team care - How well work within and outside the health center complement each other - How well staff training supports continuity of care. - How well health center integration is reflected in all documents/processes/activities | No or minimal integration | | | Fair level of integration | | | Good level of integration | | | Very good level of integration | | |
|  | **0** | **1** | **2** | **3** | **4** | **5** | **6** | **7** | **8** | **9** | **10** | **11** |
|  | **Justification:** | | | | | | | | | | | |
